# Whole-body biodistribution and radiation dosimetry of [^18^F]PR04.MZ: a new PET radiotracer for clinical management of patients with movement disorders

**DOI:** 10.1186/s13550-021-00873-9

**Published:** 2022-01-10

**Authors:** Wencke Lehnert, Patrick J. Riss, Ana Hurtado de Mendoza, Sandra Lopez, Gonzalo Fernandez, Marcelo Ilheu, Horacio Amaral, Vasko Kramer

**Affiliations:** 1 Department of Nuclear Medicine, University Medical Center Hamburg, Hamburg, Germany; 2 Department of Chemistry, University of Oslo, Oslo, Norway; 3 Center for Nuclear Medicine and PET/CT Positronmed, 7501068 Providencia, Santiago Chile; 4 Positronpharma SA, Rancagua 878, 7500921 Providencia, Santiago Chile

**Keywords:** [^18^F]PR04.MZ, Dosimetry, Positron emission tomography, Dopamine transporter, Parkinson’s disease

## Abstract

**Purpose:**

[^18^F]PR04.MZ is a new PET imaging agent for dopamine transporters, providing excellent image quality and allowing for the evaluation of patients with movement disorders such as Parkinson’s disease. The objective of this study was to evaluate the biodistribution and radiation dosimetry of [^18^F]PR04.MZ by serial PET imaging.

**Methods:**

Six healthy subjects (*n* = 3 males, *n* = 3 females) were enrolled in this study. A series of 14 whole-body PET/CT scans were acquired until 5.5 h post-injection of 200 ± 11 MBq of [^18^F]PR04.MZ. After rigid co-registration, volumes of interest were outlined either on CT or PET images. Time-integrated activity coefficients were calculated for selected source organs. Organ absorbed doses, and the effective dose were calculated using IDAC-Dose 2.1.

**Results:**

Physiological uptake of [^18^F]PR04.MZ was mainly observed in the striatum, brain, liver, gall bladder, intestine, red marrow and cortical bone. [^18^F]PR04.MZ was primarily excreted via hepatobiliary clearance and, to a lower extent, via renal clearance. The normalized absorbed doses were highest in gall bladder wall (32.2 ± 6.4 µGy/MBq), urinary bladder wall (27.2 ± 4.5 µGy/MBq), red marrow (26.5 ± 1.4 µGy/MBq), cortical bone surface (26.3 ± 2.5 µGy/MBq), liver (22.5 ± 1.8 µGy/MBq) and kidneys (21.8 ± 1.1 µGy/MBq). The effective dose according to ICRP 60 and 103 was 16.3 ± 1.1 and 16.6 ± 1.5 µSv/MBq, respectively.

**Conclusion:**

[^18^F]PR04.MZ has a favourable dosimetry profile, comparable to those of other ^18^F-labelled PET tracers, and is suitable for larger clinical applications.

*Trial registration* CEC SSM Oriente, Santiago, Chile, permit 20140520.

## Introduction

The presynaptic dopamine transporter (DAT) modulates the extracellular dopamine concentration in the brain. DAT availability is an important imaging biomarker of different disease states, such as movement disorders, attention deficit hyperactivity disorder (ADHD) and drug addiction [1, 2]. Particularly for Parkinsonian syndromes (PS), including Parkinson’s disease (PD), multiple system atrophy and progressive supranuclear palsy, characterized by degeneration of dopaminergic, nigro-striatal neurons, DAT is an excellent target for diagnostic imaging [3, 4].

For disease management, DAT imaging is used to differentiate essential tremor from tremor due to PS for which the Single Photon Emission Computed Tomography (SPECT) tracer DaTscan™ (Ioflupane I-123), approved by the FDA and EMA, is the standard of care [5]. Although Positron Emission Tomography (PET) is the superior imaging technique, no PET tracer has been approved for the same purpose to date which might be related to higher costs and complicated quantification of earlier generations of DAT tracers.

DAT PET imaging with ^18^F-labelled tracers such as [^18^F]FP-CIT, [^18^F]FE-CNT, [^18^F]FE-PE2I, [^18^F]LBT-999 is a viable alternative when precise quantification for clinical research is necessary or where DaTscan™ is not available [6–10].

[^18^F]PR04.MZ is a new, innovative PET imaging agent with an improved affinity and selectivity profile, which further allows for quantification of extra-striatal DAT, for example in the substantia nigra pars compacta [11, 12]. The pharmacokinetic evaluation of [^18^F]PR04.MZ showed very high specific uptake in striatal and midbrain regions, excellent imaging contrast and robust quantification outcomes [13]. Following the clinical translation, the diagnostic potential of this new tracer was highlighted by the excellent imaging properties in a case study of Holmes Tremor [14] and the clinical validation for detection of nigro-striatal degeneration in patients under evaluation for movement disorders [15].

The aim of this study was to evaluate the long-term biodistribution and dosimetry of [^18^F]PR04.MZ PET/CT in healthy controls to support clinical application in larger groups of patients.

## Materials and methods

### Subjects

The study was approved by the regional ethics committee board (CEC SSM Oriente, permit 20140520), and written informed consent has been obtained from all participants. We included six healthy controls (HC) (3 males, 3 females) in the study with a mean age of 28 ± 3 years, mean body mass index of 23.9 ± 6.3 and injected activities of 200 ± 11 MBq. Detailed demographics are shown in Table 1. All subjects were of normal health and free of any medical condition at the time of the scans as determined by medical examination and questionnaire. One subject underwent cholecystectomy prior to inclusion. Subjects fasted on the day of imaging until the end of the study. [^18^F]PR04.MZ was produced under GMP-compliant conditions as previously described [13].

**Table 1:** Physical characteristics of included subjects

Subject number	Sex	Age (years)	Height (m)	Weight (kg)	BMI (kg/m^2^)	Activity Injected (mCi)	Activity Injected (MBq)
1	M	29	1.71	50	17.1	5.58	206.5
2	M	29	1.75	66	21.6	5.18	191.7
3	M	23	1.72	74	25.0	5.09	188.3
4	F	27	1.61	60	23.1	5.13	189.8
5	F	29	1.59	53	21.0	5.70	210.9
6	F	30	1.65	97	35.6	5.74	212.4
Mean ± SD	NA	28 ± 3	1.67 ± 0.06	67 ± 17	23.9 ± 6.3	5.40 ± 0.30	200 ± 11

### PET/CT imaging

For all participants, a series of 14 whole-body (WB) PET/CT scans (Biograph Vision: *n* = 3 subjects, Biograph mCT Flow: *n* = 3 subjects, both Siemens, Erlangen, Germany) covering head to mid-thigh (10–11 bed positions) was acquired over the course of 5.5 h and in three separate sessions as follows: Session 1 consisting of ten consecutive PET scans from 0 to 120 min post-injection (p.i.) (4 × 30 s/bed position, 4 × 60 s/bed position, and 2 × 120 s/bed position), break 1 from 120 to 180 min p.i., session 2 consisting of two consecutive PET scans from 180 to 225 min p.i. (270 s/bed position), break 2 from 225 to 285 min p.i., session 3 consisting of two consecutive PET scans from 285 to 330 min p.i. (270 s/bed position). Subjects were asked to void their bladder after each imaging session. A low-dose CT scan was performed for attenuation correction and co-registration prior to each PET imaging session. PET data were corrected for random coincidences, normalization, attenuation, scatter, and dead-time losses. The data were reconstructed using an ordinary Poisson ordered subset expectation maximization (OP-OSEM) 3D iterative algorithm (Vision: 9 iterations, 5 subsets, matrix: 220 × 220; mCT Flow: 2 iterations and 21 subsets, matrix: 200 × 200) applying time-of-flight (ToF) and point-spread function (PSF) modelling with post-reconstruction smoothing (Gaussian, 4 mm full-width at half-maximum).

To yield quantitative images in units of Bq/ml, a cross-calibration of the PET/CT scanner and dose calibrator was performed every three months according to the recommendations of the manufacturer. A cylindrical uniformity phantom filled with an activity of approx, 60 MBq at the time of each PET scan was used. The deviation between original and previous efficiency calibration factor had to be less than 5%. In addition, daily quality control of the PET/CT scanner was performed and passed before every study.

### Image processing and analysis

Image processing and analysis was performed using PMOD v3.4 (PMOD Technologies, Zurich, Switzerland). Volumes of interest (VOI) were manually drawn on CT images for relevant organs (brain, kidneys, lungs, liver, pancreas, spleen, stomach, left and right colon, skeleton and femur, total heart, left ventricle and left ventricle content, red marrow in the lumbar vertebrae L2-L4) and the part of the total body in the field of view. The VOIs were transferred to the corresponding, co-registered PET images with minor manual adjustment if needed. For gall bladder, urinary bladder, parotid and submaxillary glands, VOIs were outlined by threshold-based segmentation on each individual PET scan.

Not all VOIs were directly used as source organs. Total activity in red marrow was calculated using the activity from red marrow VOIs outlined for L2-L4 vertebrae assuming that L2-L4 constitute 60% of total lumbar vertebrae volume and the lumbar vertebrae contain 12.3% of the total red marrow mass [16]. Activity in the heart wall was calculated as activity in the left ventricle subtracted by the activity in the left ventricle content and likewise the activity in the heart content was estimated from the total heart activity subtracted by the activity in the heart wall.

For calculation of total bone activity, the femora were excluded from the skeleton VOI. As the segmented VOI for the skeleton did not include the legs and with the legs representing 33.6% of total bone mass, the obtained activity was scaled by a factor of 1.506 (1/0.664) [16, 17]. The activity of the red marrow was subtracted. The total bone activity was then distributed between cortical bone mineral surface (80%) and trabecular bone mineral surface (20%) according to [17].

Finally, non-decay corrected time-activity-curves (TACs) were generated for all source organs and the total body VOI.

### Dosimetry analysis

The TACs for all source organs were imported as externally measured organs into the dosimetry software suite QDOSE (ABX-CRO advanced pharmaceutical services Forschungsgesellschaft m.b.H, Dresden, Germany). All TACs were fitted depending on the degree of correlation to a mono-, bi- or tri-exponential function, excluding initial time points if necessary for the fit. The time-integrated activity (also called cumulated activity) for each source organ was determined by calculating the area under the curve of the TAC using numerical integration until the last imaging time point and adding the area under the fitted curve extrapolated from this point until infinity.

The time-integrated activity for the total body was calculated by scaling the time-integrated activity of the total body VOI with the scaling factor comprising the injected activity divided by the activity at time = 0 for the curve fit of the total body VOI. The time-integrated activity in the remainder body was then automatically calculated by subtracting the time-integrated activities in all source organs from the total body time-integrated activity.

The time-integrated activity coefficient (TIAC) (also called residence time) was calculated for each source organ as the time-integrated activity divided by the administered activity.

The absorbed organ doses and effective dose calculations were performed using IDAC-Dose 2.1 [18] integrated in QDOSE. Standard phantom organ masses were utilized for dose calculation and no adaption to individual organ masses was performed. The effective dose (ED) was calculated according to International Commission on Radiation Protection (ICRP) guidelines 60 and 103 [19, 20]. The absorbed doses to the salivary glands were determined using the spherical model [21] assuming organ masses of 25.0 g and 12.5 g for a single parotid gland and submandibular gland, respectively [17].

## Results

The administered activity of [^18^F]PR04.MZ was 200 ± 11 MBq (mean ± SD, range 188–212 MBq), there were no adverse or clinically detectable pharmacologic effects (injected mass was < 1.0 ug) in any of the six subjects and no significant changes in vital signs were observed.

### Biodistribution

**Fig. 1:**
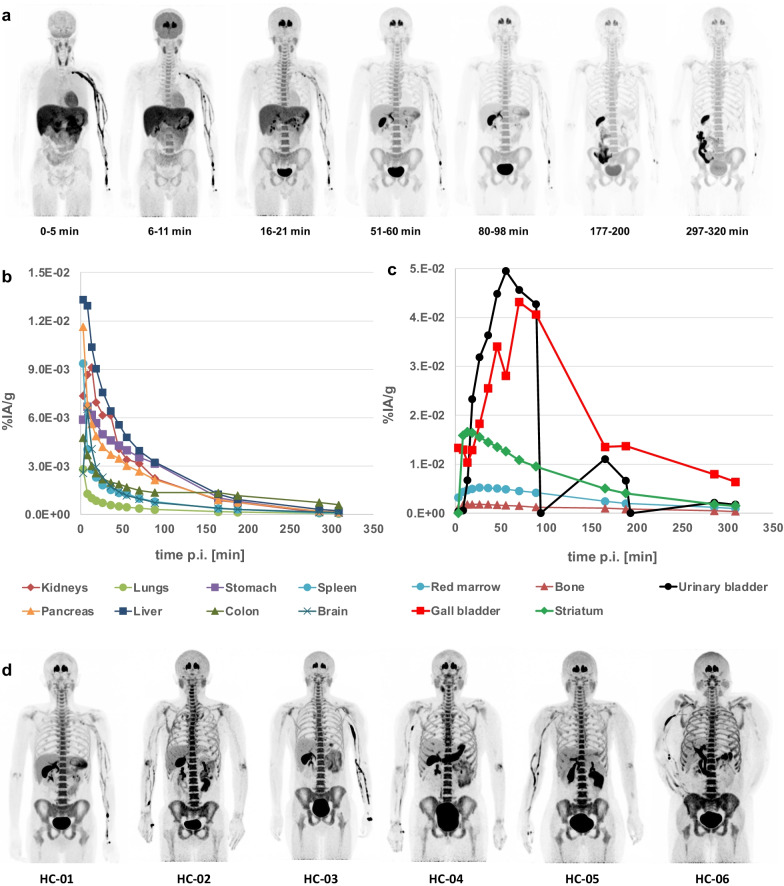
**a** Representative maximum-intensity projections for a series of PET images over 320 min after injection of [^18^F]PR04.MZ, **b** representative TACs for subject 1 and for major organs expressed as per cent injected activity (%IA/g), **c** representative TACs for urinary bladder, gall bladder, striatum, red marrow and bone, **d** representative maximum-intensity projections of PET images of all HCs approximately 60–90 min after injection of [^18^F]PR04.MZ

Physiological uptake of [^18^F]PR04.MZ was observed in the striatum, brain, heart, parotid and submaxillary glands, thyroid, liver, gall bladder, intestine, red marrow and cortical bone (Fig. 1a). The distribution pattern of [^18^F]PR04.MZ for different organs can be distinguished according to different kinetic profiles. Initial uptake and fast clearance within 30–40 min p.i. were observed for major organs including heart, stomach, liver, lungs, kidneys, spleen and pancreas. Higher, specific uptake and prolonged retention of [^18^F]PR04.MZ were observed for tissues with high DAT expression such as striatum and red marrow. For both, striatum and red marrow, peak uptake of 0.017 and 0.005%IA/g was observed around 15 and 25 min p.i., respectively. [^18^F]PR04.MZ was primarily cleared via the hepatobiliary pathway and, to a lower extent, via the renal pathway. Accordingly, a high uptake was observed in the urinary bladder, gall bladder and colon contents at very late time points (Fig. 1b).

When considering bone and red marrow activity uptake, the accumulation of [^18^F]PR04.MZ in red marrow was about 2.9 times higher than for cortical and trabecular bone and followed the same kinetic profile as for the striatal region in the brain (Fig. 1c).

### Dosimetry

TIACs for all source organs and individual subjects, as well as mean and standard deviation, are shown in Table 2. Highest mean TIACs were calculated for cortical bone with 0.290 h, red marrow (0.208 h), liver (0.138 h), urinary bladder content (0.101 h), trabecular bone (0.072 h), colon contents (0.062 h), lungs (0.048 h), brain (0.045 h), gallbladder content (0.028 h) and kidneys (0.027 h).

**Table 2:** Time-integrated activity coefficients (TIAC) for [^18^F]PR04.MZ for all subjects and source organs

Organ	TIAC (MBq h/MBq)								
	HC-01 (M)	HC-02 (M)	HC-03 (M)	HC-04 (F)	HC-05 (F)	HC-06 (F)	Mean	StDev	CoV (%)
Brain^a^	5.59E−02	4.80E−02	4.00E−02	3.87E−02	4.61E−02	3.82E−02	4.45E−02	6.90E−03	15.5
Cortical bone mineral surface^a^	2.97E−01	2.86E−01	3.53E−01	2.82E−01	2.13E−01	3.08E−01	2.90E−01	4.55E−02	15.7
Gallbladder content^a^	3.34E−02	3.98E−02	3.93E−02	3.23E−02	1.95E−02	5.77E−03^b^	2.83E−02	1.33E−02	46.8
Heart contents	1.95E−02	2.34E−02	2.31E−02	1.73E−02	1.61E−02	2.29E−02	2.04E−02	3.21E−03	15.7
Heart wall^a^	7.63E−03	8.15E−03	7.47E−03	6.72E−03	4.96E−03	5.76E−03	6.78E−03	1.22E−03	18.0
Kidneys^a^	2.85E−02	2.95E−02	2.59E−02	2.36E−02	2.54E−02	2.60E−02	2.65E−02	2.16E−03	8.1
Left colon contents^a^	7.50E−02	8.30E−02	5.99E−02	6.34E−02	4.61E−02	4.44E−02	6.20E−02	1.54E−02	24.8
Liver^a^	1.42E−01	1.45E−01	1.45E−01	1.41E−01	1.43E−01	1.12E−01	1.38E−01	1.28E−02	9.3
Lungs^a^	6.31E−02	4.01E−02	4.90E−02	3.82E−02	4.42E−02	5.25E−02	4.79E−02	9.19E−03	19.2
Pancreas^a^	1.17E−02	2.99E−03	4.19E−03	6.86E−03	6.15E−03	5.96E−03	6.31E−03	3.00E−03	47.6
Parotid glands	4.18E−03	5.44E−03	4.60E−03	5.72E−03	5.41E−03	2.27E−03	4.60E−03	1.28E−03	27.9
Red marrow^a^	2.44E−01	2.38E−01	2.08E−01	1.74E−01	1.92E−01	1.92E−01	2.08E−01	2.78E−02	13.4
Right colon contents^a^	7.50E−02	8.30E−02	5.99E−02	6.34E−02	4.61E−02	4.44E−02	6.20E−02	1.54E−02	24.8
Spleen^a^	4.40E−03	4.19E−03	7.09E−03	2.99E−03	2.88E−03	5.57E−03	4.52E−03	1.60E−03	35.5
Stomach contents^a^	4.11E−02	1.89E−02	1.17E−02	3.43E−02	1.53E−02	4.92E−03	2.10E−02	1.39E−02	66.0
Submaxillary glands	3.01E−03	2.66E−03	3.16E−03	2.70E−03	3.01E−03	2.85E−03	2.90E−03	1.96E−04	6.8
Total body^a^	2.19E + 00	2.40E + 00	2.28E + 00	2.28E + 00	2.24E + 00	2.32E + 00	2.29E + 00	7.15E−02	3.1
Trabecular bone mineral surface^a^	7.41E−02	7.14E−02	8.82E−02	7.06E−02	5.33E−02	7.69E−02	7.24E−02	1.13E−02	15.6
Urinary bladder content^a^	9.63E−02	1.19E−01	1.19E−01	1.14E−01	1.12E−01	4.54E−02	1.01E−01	2.85E−02	28.2

^a^Used as input for dose calculations in IDAC 2.1

^b^Subject underwent cholecystectomy prior to inclusion—activity uptake from bile duct

The normalized absorbed doses for all major organs are shown in Table 3. Highest values were obtained in the gall bladder wall (32.2 ± 6.4 µGy/MBq), followed by urinary bladder wall (27.2 ± 4.5 µGy/MBq), red marrow (26.5 ± 1.4 µGy/MBq), cortical bone surface (26.3 ± 2.5 µGy/MBq), liver (22.5 ± 1.8 µGy/MBq) and kidneys (21.8 ± 1.1 µGy/MBq). The effective dose according to ICRP 60 and 103 was 16.3 ± 1.1 and 16.6 ± 1.5 µSv/MBq, respectively.

**Table 3:** Normalized absorbed doses and effective doses for [^18^F]PR04.MZ for all subjects and selected target organs calculated with IDAC-Dose 2.1

	HC-01 (M)	HC-02 (M)	HC-03 (M)	HC-04 (F)	HC-05 (F)	HC-06 (F)	Mean	StDev	CoV (%)
*Organ*	*Normalised absorbed dose (mGy/MBq)*								
Adrenals	1.43E−02	1.51E−02	1.40E−02	1.94E−02	1.84E−02	1.72E−02	1.64E−02	2.26E−03	13.8
Brain	1.18E−02	1.08E−02	9.72E−03	1.05E−02	1.14E−02	1.09E−02	1.09E−02	7.22E−04	6.7
Breasts	6.71E−03	7.39E−03	6.83E−03	8.48E−03	8.74E−03	9.12E−03	7.88E−03	1.03E−03	13.1
Colon	1.69E−02	1.87E−02	1.56E−02	1.95E−02	1.73E−02	1.67E−02	1.75E−02	1.42E−03	8.1
Cortical bone surface	2.40E−02	2.42E−02	2.59E−02	2.81E−02	2.51E−02	3.04E−02	2.63E−02	2.50E−03	9.5
Eye lenses	5.58E−03	6.18E−03	5.91E−03	7.03E−03	7.12E−03	7.81E−03	6.61E−03	8.50E−04	12.9
Gallbladder wall	3.30E−02	3.65E−02	3.47E−02	3.83E−02	3.01E−02	2.04E−02^a^	3.22E−02	6.42E−03	20.0
Heart wall	1.16E−02	1.15E−02	1.08E−02	1.23E−02	1.12E−02	1.18E−02	1.15E−02	5.13E−04	4.4
Kidneys	2.12E−02	2.22E−02	1.98E−02	2.25E−02	2.25E−02	2.23E−02	2.18E−02	1.07E−03	4.9
Left colon wall	1.83E−02	1.99E−02	1.60E−02	2.16E−02	1.87E−02	1.83E−02	1.88E−02	1.87E−03	9.9
Liver	2.18E−02	2.23E−02	2.16E−02	2.48E−02	2.42E−02	2.01E−02	2.25E−02	1.75E−03	7.8
Lungs	1.50E−02	1.32E−02	1.37E−02	1.50E−02	1.58E−02	1.74E−02	1.50E−02	1.51E−03	10.0
Muscle	7.25E−03	8.40E−03	7.87E−03	1.01E−02	1.02E−02	1.08E−02	9.10E−03	1.45E−03	15.9
Oesophagus wall	1.10E−02	1.15E−02	1.10E−02	1.25E−02	1.25E−02	1.32E−02	1.20E−02	9.14E−04	7.6
Ovaries	0.00E + 00	0.00E + 00	0.00E + 00	1.56E−02	1.58E−02	1.43E−02	1.52E−02	8.14E−04	5.3
Pancreas	2.59E−02	1.78E−02	1.76E−02	2.16E−02	1.91E−02	1.74E−02	1.99E−02	3.33E−03	16.7
Pituitary gland	9.50E−03	9.72E−03	9.09E−03	1.24E−02	1.24E−02	1.35E−02	1.11E−02	1.88E−03	16.9
Prostate	1.41E−02	1.69E−02	1.62E−02	0.00E + 00	0.00E + 00	0.00E + 00	1.57E−02	1.46E−03	9.3
Rectosigmoid colon wall	1.08E−02	1.28E−02	1.22E−02	1.44E−02	1.45E−02	1.27E−02	1.29E−02	1.40E−03	10.8
Red marrow	2.57E−02	2.61E−02	2.46E−02	2.69E−02	2.72E−02	2.86E−02	2.65E−02	1.38E−03	5.2
Right colon wall	1.84E−02	2.05E−02	1.70E−02	1.99E−02	1.74E−02	1.72E−02	1.84E−02	1.49E−03	8.1
SI wall	1.11E−02	1.23E−02	1.10E−02	1.51E−02	1.43E−02	1.34E−02	1.29E−02	1.69E−03	13.1
Skin	5.08E−03	5.95E−03	5.53E−03	6.88E−03	7.05E−03	7.44E−03	6.32E−03	9.38E−04	14.8
Spleen	1.17E−02	1.15E−02	1.30E−02	1.17E−02	1.08E−02	1.29E−02	1.19E−02	8.55E−04	7.2
Stomach wall	2.45E−02	1.80E−02	1.45E−02	2.26E−02	1.62E−02	1.26E−02	1.81E−02	4.65E−03	25.7
Testes	5.88E−03	7.22E−03	6.63E−03	0.00E + 00	0.00E + 00	0.00E + 00	6.58E−03	6.72E−04	10.2
Thymus	9.25E−03	9.89E−03	9.47E−03	1.11E−02	1.14E−02	1.23E−02	1.06E−02	1.21E−03	11.5
Thyroid	7.81E−03	8.64E−03	8.21E−03	9.66E−03	9.94E−03	1.08E−02	9.18E−03	1.14E−03	12.5
Ureters	1.28E−02	1.45E−02	1.33E−02	1.78E−02	1.72E−02	1.66E−02	1.54E−02	2.12E−03	13.8
Urinary bladder wall	2.44E−02	2.95E−02	2.89E−02	3.07E−02	3.04E−02	1.92E−02	2.72E−02	4.53E−03	16.7
Uterus/Cervix	0.00E + 00	0.00E + 00	0.00E + 00	1.91E−02	1.92E−02	1.54E−02	1.79E−02	2.17E−03	12.1
*ICRP Standard*	*Normalised effective dose (mSv/MBq)*								
ED ICRP 60	1.75E−02	1.73E−02	1.61E−02	1.65E−02	1.54E−02	1.47E−02	1.63E−02	1.08E−03	6.7
ED ICRP 103	1.59E−02	1.60E−02	1.47E−02	1.91E−02	1.75E−02	1.64E−02	1.66E−02	1.52E−03	9.2

^a^Subject underwent cholecystectomy prior to inclusion

## Discussion

In this study, six healthy subjects were enrolled and whole-body PET/CT scans were acquired over 5.5 h post-injection to evaluate the biodistribution and dosimetry of [^18^F]PR04.MZ.

While the initial uptake of [^18^F]PR04.MZ in tissue of the brain, heart, parotid and submaxillary glands and thyroid is related to perfusion and cleared very rapidly, uptake in the liver and subsequently in gallbladder, intestine as well as kidneys and urinary bladder can be attributed to the main excretion pathways of the tracer. Also, a small amount of the tracer was retained at the injection side and in the ascending vein, depending on the volume of saline used to flush the injection port, but the effect on the dose calculations is considered negligible. In contrast, prolonged retention of [^18^F]PR04.MZ was observed in the striatum and red marrow due to specific binding to DAT, which has been observed for other DAT PET tracers to a similar extent [22].

In agreement with the observed biodistribution, the highest absorbed dose was received by the gall bladder wall (32.2 ± 6.4 µGy/MBq), urinary bladder wall (27.2 ± 4.5 µGy/MBq), red marrow (26.5 ± 1.4 µGy/MBq), cortical bone surface (26.3 ± 2.5 µGy/MBq) and liver (22.5 ± 1.8 µGy/MBq) in 5/6 subjects. The subject which underwent cholecystectomy prior to study inclusion still showed activity uptake in the biliary ductus. Due to a significant contribution of the liver to the gall bladder dose, the absorbed dose to the gall bladder wall was only 40% lower than the median dose across all subjects without relevant impact on effective dose calculation.

For [^18^F]FP-CIT and [^18^F]FE-PE2I, the urinary bladder wall had the highest absorbed dose with mean values of 58.6 µGy/MBq [22] and 119 µGy/MBq [23] which, compared to the current study, may in part be related to some differences in methodology to calculate the TIAC for urinary bladder content, as well as differences in the dose calculation programme. Another contributing factor may be binding of these tracers to serotonin transporters (SERT) and norepinephrine transporters (NET) expressed on smooth muscle cells of the urinary bladder wall [24]. Since [^18^F]PR04.MZ provides a higher selectivity for DAT [11], the contribution of SERT and NET binding to the absorbed dose for the urinary bladder wall may be lower. In addition, no polar metabolites other than [^18^F]Fluoride have been described for [^18^F]PR04.MZ which would be primarily cleared via the kidneys and bladder [13].

It can further be assumed that the absorbed dose to bone surface was higher in the current study because cortical and trabecular bone were explicitly used as source organs compared to other ^18^F-labelled DAT PET tracers where bone was included in the remainder body [22, 23]. It appears that there is a significant contribution of hydroxyapatite-bound [^18^F]Fluoride to the absorbed dose to bone surface since the dose contribution from red marrow tissue as source organ to bone is only 12%.

Similarly, gallbladder content was only used as a source organ in this study, resulting in a higher absorbed dose as compared to [^18^F]FE-PE2I [23] and [^18^F]FP-CIT [22]. A similar absorbed dose to the red marrow was observed for [^18^F]FE-PE2I with 25 µGy/MBq [23] compared to 26.5 µGy/MBq for the investigated [^18^F]PR04.MZ. In both cases, red marrow was included as a source organ and in addition, both used IDAC (IDAC-Dose 2.0 in [23] and IDAC-Dose 2.1 the current work) for dose calculation applying the same assumptions for red marrow dose calculation. In the dosimetry for [^18^F]FP-CIT, red marrow was not included as source organ and hence the red marrow absorbed dose was lower (5.11 µGy/MBq) [22].

Table 4 provides a comparison of dosimetry data for different DAT PET tracers, as well as for [^123^I]FP-CIT. The effective dose of [^18^F]PR04.MZ calculated with IDAC-Dose 2.1 according to ICRP 60 and 103 was 16.3 ± 1.1 µSv/MBq and 16.6 ± 1.5 µSv/MBq, respectively, which is within the standard range of 15–30 µSv/MBq observed for the vast majority of ^18^F-labelled PET tracers [25]. The effective dose for [^18^F]FE-PE2I, calculated with IDAC 2.0 according to ICRP 60, was higher with 23 µSv/MBq [23]. This should be mainly related to a higher TIAC for the remainder body with bone surface not used as a separate source organ [23] and the very low tissue weighting factor for bone surface of 0.01 in ICRP 60 [19] and also ICRP 103 [20]. For [^18^F]FP-CIT, the effective dose equivalent according to ICRP 26 [26] was calculated using MIRDOSE 3 (a predecessor of OLINDA/EXM) resulting in a lower effective dose of 12.0 µSv/MBq [22]. Despite the different radionuclide, the reported effective dose (ICRP 60) for [^123^I]FP-CIT, the widely used DaTscan™ SPECT tracer, is also in that range with 23.5 µSv/MBq [27].

**Table 4:** Human dosimetry data for different DAT PET tracers compared to [^123^I]FP-CIT

	[^18^F]PR04.MZ	[^18^F]FE-PE2I	[^18^F]FP-CIT	[^123^I]FP-CIT
Highest organ absorbed dose [mGy/MBq]	Gallbladder wall: 3.22E−02	Urinary bladder wall: 1.19E−01	Urinary bladder wall: 5.86E−02	Urinary bladder wall: 5.35E−02
2^nd^ highest organ absorbed dose [mGy/MBq]	Urinary bladder wall: 2.72E−02	Liver: 4.6E−02	Lungs: 1.92E−02	Lungs: 4.25E−02
3^rd^ highest organ absorbed dose [mGy/MBq]	Red marrow: 2.65E−02	Pancreas: 3.1E−02	Liver: 1.86E−02	Lower large intestine: 4.24E−02
Effective dose equivalent—ICRP 26 [mSv/MBq]	–	–	1.20E−02	2.44E−02
Effective dose—ICRP 60 [mSv/MBq]	1.63E−02	2.3E−02	–	2.35E−02
Effective dose—ICRP 103 [mSv/MBq]	1.66E−02	–	–	–
Dose calculation software	IDAC-Dose 2.1	IDAC 2.0	MIRDOSE 3	MIRDOSE 3.1
Reference	This study	[22]	[23]	[27]

In the current study, we have also performed dose calculations using IDAC-Dose 1.0 based on the Cristy-Eckerman phantom series as MIRDOSE [28] and OLINDA/EXM 1.1 [29] and the ICRP 60 and 26 standards for effective dose calculation as it is also available in QDOSE. The absorbed doses were higher by a factor of 2 for the urinary bladder wall with 56.9 µGy/MBq and upper/lower large intestine wall with 45.9 and 29.7 µGy/MBq, respectively. Consequently, the effective dose was also higher with 21.2 µSv/MBq (ICRP 60) and 22.6 µSv/MBq (ICRP 26) related to the tissue weighting factors of individual organs and remainder body.

However, IDAC-Dose 2.1 based on the ICRP adult reference computational phantoms [30] and the updated tissue weighting factors according to ICRP publication 103 can be considered the current standard and, hence, have been used to assess and report dose estimates here. One should keep in mind that comparisons to previous publications can be influenced by these differences in dose calculation software and the continuous evolution of calculation protocols. Based on the results of the current study, a standard injection of 185 MBq [^18^F]PR04.MZ would result in an effective dose of approximately 3 mSv, far below the most strict dose limit of 10 mSv used in some European countries [31].

## Conclusion

[^18^F]PR04.MZ has a favourable dosimetry profile which is comparable to other ^18^F-labelled PET tracers and would result in an average effective dose of 16.3 ± 1.1 µSv/MBq. This is within the standard range of 15–30 µSv/MBq observed for the majority of ^18^F-labelled PET tracers and a standard injection of 185 MBq [^18^F]PR04.MZ would result in an effective dose of approximately 3 mSv. In addition, [^18^F]PR04.MZ showed high specific uptake in striatal and midbrain regions [13] and showed very promising results for the evaluation of the dopaminergic, nigro-striatal integrity in patients with movement disorders such as Holmes Tremor [14] PS [15]. In combination with the excellent imaging properties, the dosimetry profile of [^18^F]PR04.MZ supports clinical applications in larger groups of patients being evaluated for movement disorders.

## Data Availability

The raw data and datasets generated during and/or analysed during the current study are available from the corresponding author on reasonable request.
